# Supplementary material for: D-Mannose treats obesity by increasing adipose Treg cells and suppressing gut Firmicutes
Source: bioRxiv. 2026 Aug 14:2026.08.10.743951. Preprint. [Version 1] doi: 10.64898/2026.08.10.743951 (PMC13640646; doi:10.64898/2026.08.10.743951)
Supplement: Supplementary file 1 [file NIHPP2026.08.10.743951v1-supplement-1.pdf]

(which was not certified by peer review) is the author/funder. This article is a US Government work. It is not subject to copyright under 17 USC 105 and is also made available for use under a CC0 license.

SUPPLEMENTAL FIGURES AND TABLES

(which was not certified by peer review) is the author/funder. This article is a US Government work. It is not subject to copyright under 17 USC 105 and is also made available for use under a CC0 license.

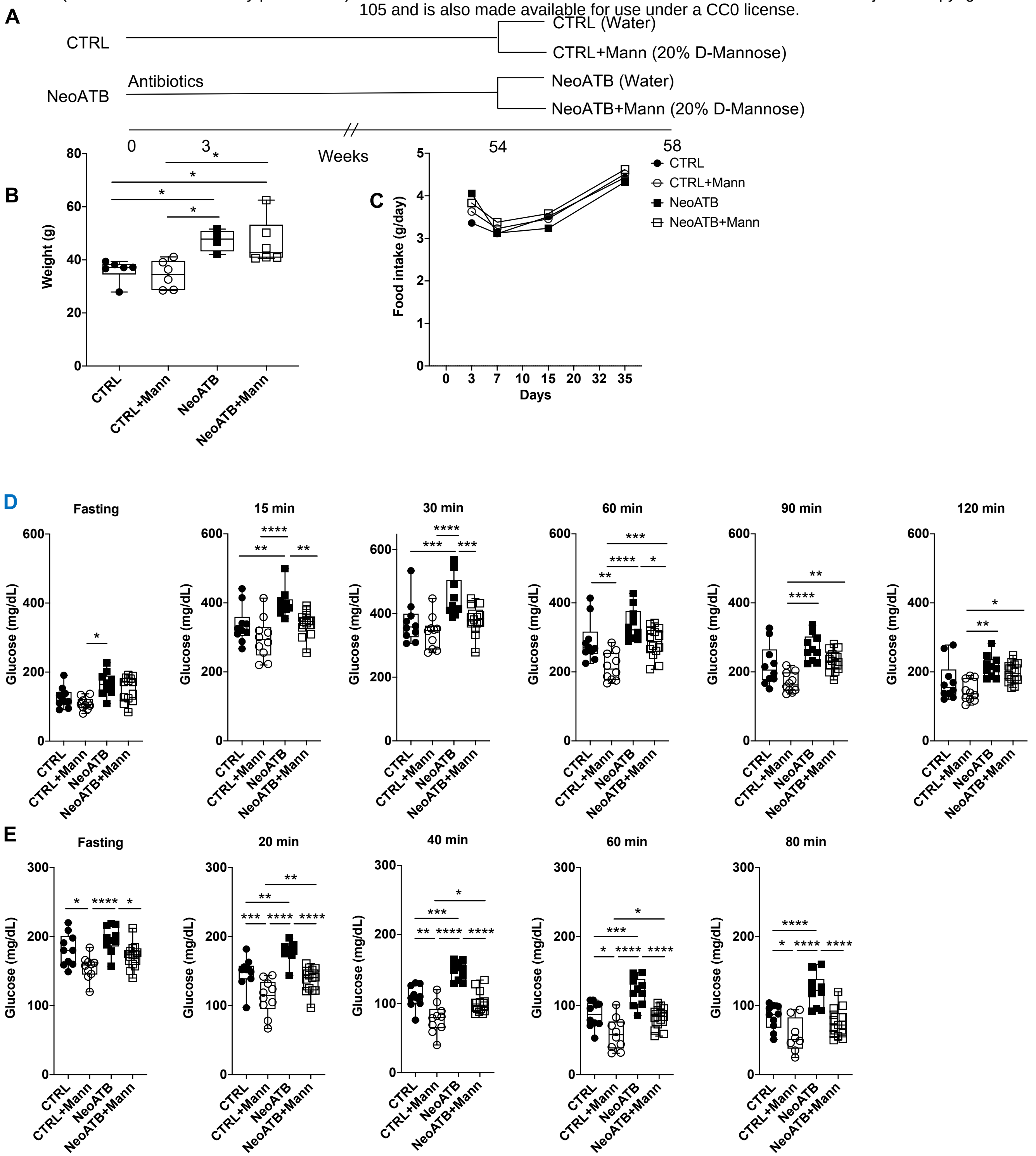

D-mannose improves metabolism of NeoATB mice

a) Immunization scheme.
b) Weight of control and obese NeoATB mice at 54 weeks of age before D-mannose treatment.
c) Food intake of Control, Control treated with D-mannose, NeoATB and NeoATB treated with D-mannose mice during interval of 35 days.
d) Glucose tolerance test of Control, Control treated with D-mannose, NeoATB and NeoATB treated with D-mannose mice.
e) Insulin tolerance test of Control, Control treated with D-mannose, NeoATB and NeoATB treated with D-mannose mice

Data in d) and e) are pooled of 2 independent experiments. Each point in figures represents one individual animal. Statistical analysis was performed using one-way ANOVA (* p<0.05; ** p<0.01; *** p<0.001; **** p<0.0001).

Supplemental Figure 1

bioRxiv preprint doi: https://doi.org/10.1101/2020.08.20.174839; this version posted August 21, 2020. The copyright holder for this preprint (which was not certified by peer review) is the author/funder. This article is a US Government work. It is not subject to copyright under 17 USC 105 and is also made available for use under a CC0 license.

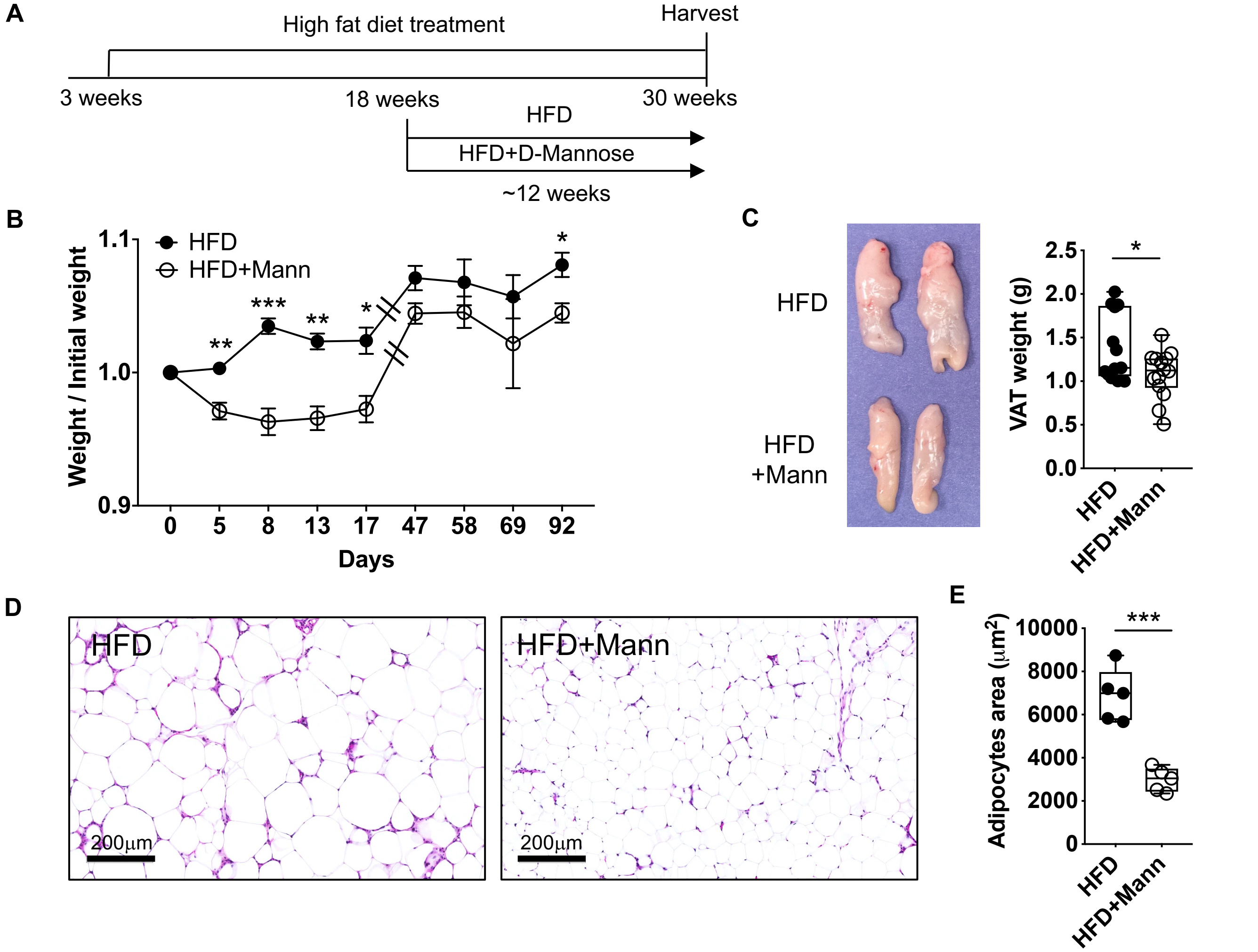

D-mannose ameliorates obesity in mice treated with a high-fat diet.

A. Immunization scheme.
B. Weight/Initial weight ratio is shown between HFD and HFD+D-Mannose treated mice.
C. Representative photograph of visceral adipose tissue (VAT) of HFD or HFD D-mannose-treated mice. Summarizing data showing the weight of VAT between HFD and HFD D-mannose-treated mice.
D. Representative H&E staining of VAT in HFD and HFD D-mannose-treated mice.
E. Summarizing data showing the size of adipocyte area between HFD and HFD D-mannose-treated mice determined using AdipoCount software.

Data are pooled from 2 independent experiments. Each point in the figures represents one individual animal. Statistical analysis was performed using a two-tailed *t-test* (* $p<0.05$; ** $p<0.01$; *** $p<0.001$).

(which was not certified by peer review) is the author/funder. This article is a US Government work. It is not subject to copyright under 17 USC 105 and is also made available for use under a CC0 license.

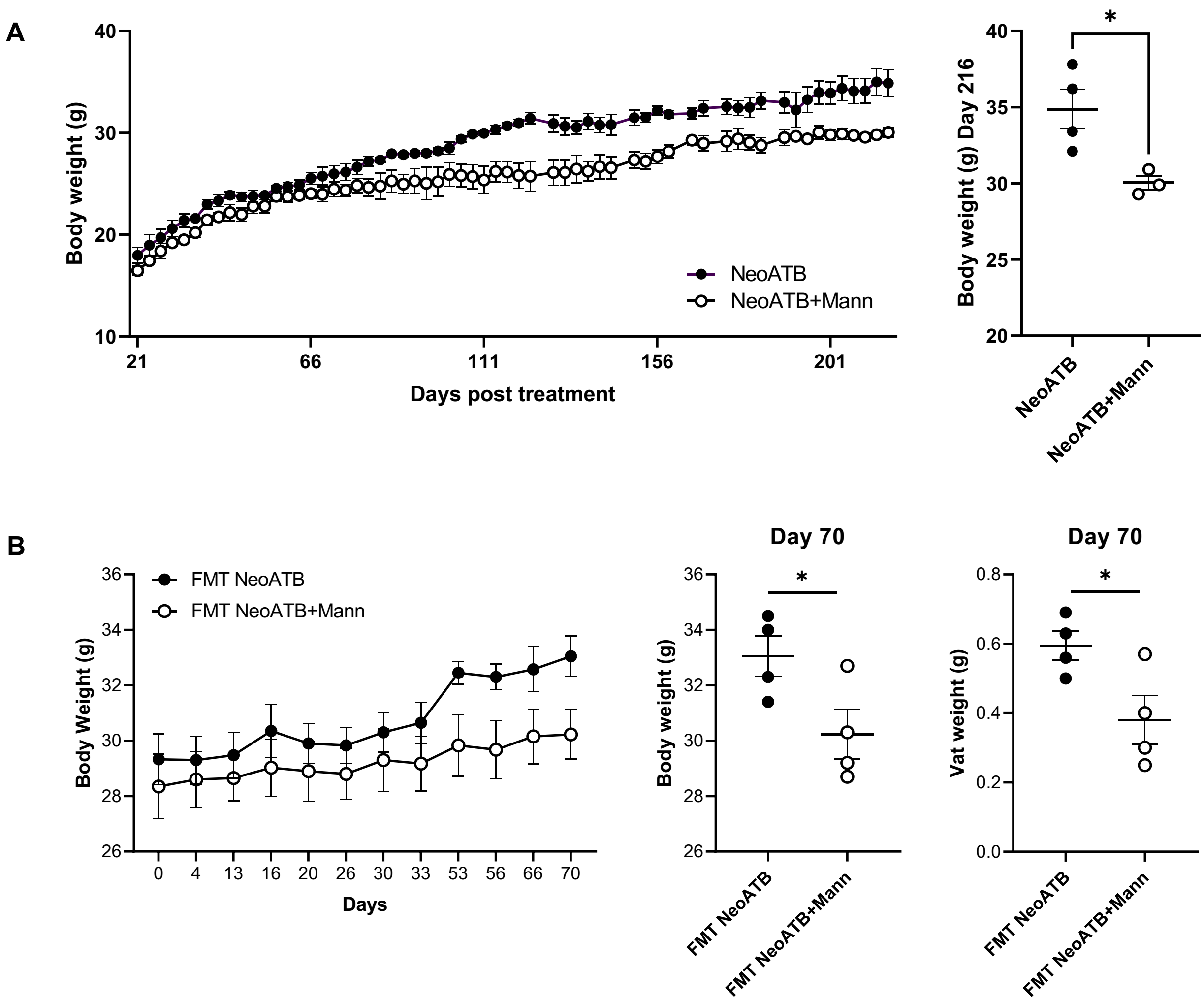

A. D-Mannose treatment can ameliorate the development of obesity in young NeoATB mice treated with antibiotics at a neonatal age. Mice were treated with vancomycin and polymyxin B during neonatal life (the first 3 weeks). After weaning, mice were separated into two groups: NeoATB (n=4) and NeoATB treated with 20% D-mannose (NeoATB+Mann; n=4) in drinking water. Body weight was monitored for 200+ days.
B. FMT transfer of microbiota from NeoATB mice treated with D-mannose ameliorated obesity in adult NeoATB mice. For the fecal microbiota transplant (FMT) experiment, we first collected fecal samples from NeoATB mice or Mannose-treated NeoATB mice, both aged 24 to 26 months. These samples were stored at -80 °C until use. On the day of the FMT, the feces were thawed, and 200 mg of the fecal material was diluted in 2 mL of phosphate-buffered saline (PBS). The mixture was then homogenized and thoroughly mixed. Next, the fecal suspension was filtered, and centrifugation was performed at 600g for 5 minutes to pellet any undissolved solid matter. The supernatant was then gavaged to the recipient mice at a dosage of 10 µL per gram of body weight, administered every other day for a total of three doses.

The data is representative of 1 experiment. Statistical analysis was performed using a two-tailed *t-test* or two-way ANOVA test (* $p<0.05$).

Supplemental Figure 3

(which was not certified by peer review) is the author/funder. This article is a US Government work. It is not subject to copyright under 17 USC
105 and is also made available for use under a CC0 license.

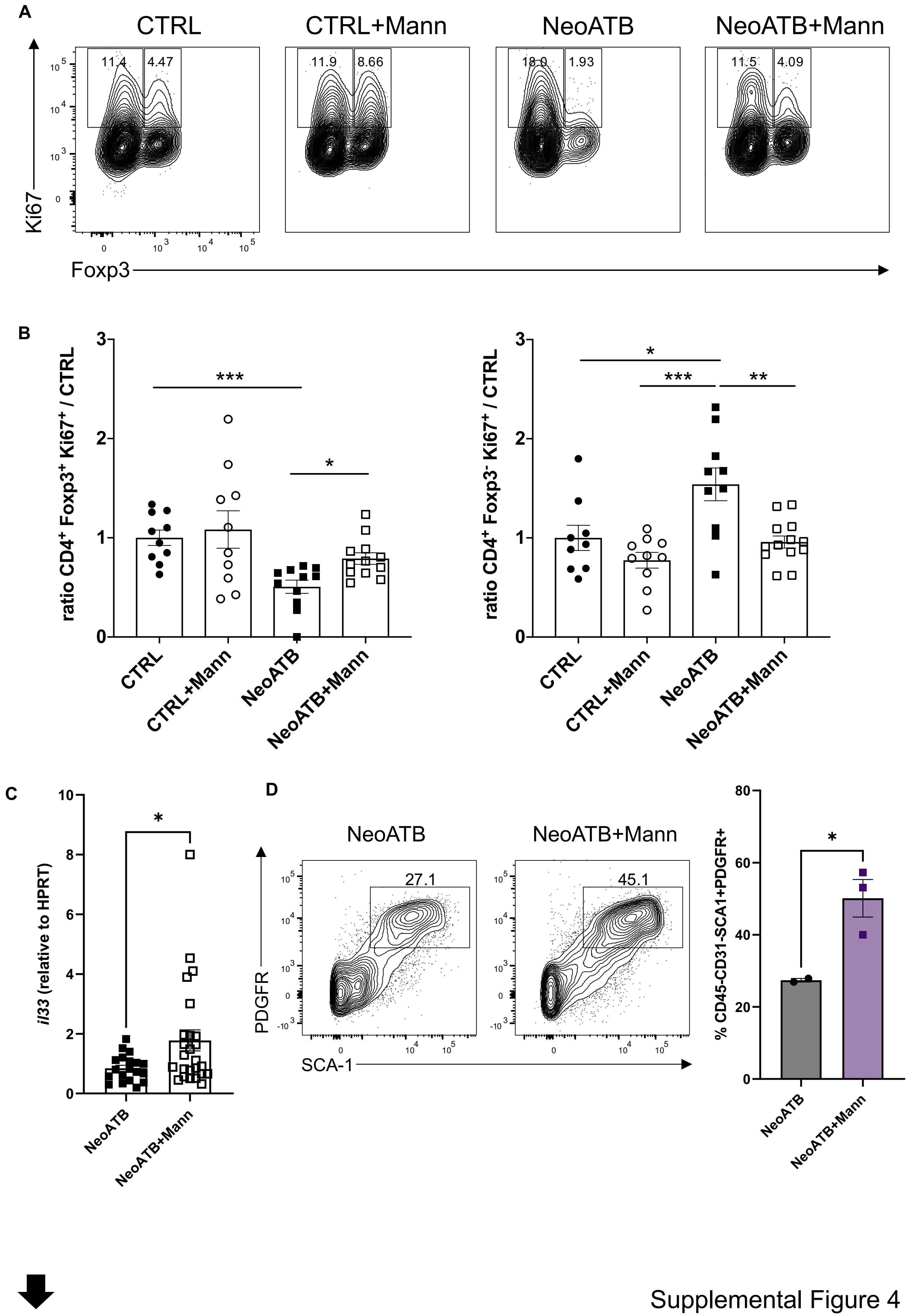

Supplemental Figure 4

(which was not certified by peer review) is the author/funder. This article is a US Government work. It is not subject to copyright under 17 USC 105 and is also made available for use under a CC0 license.

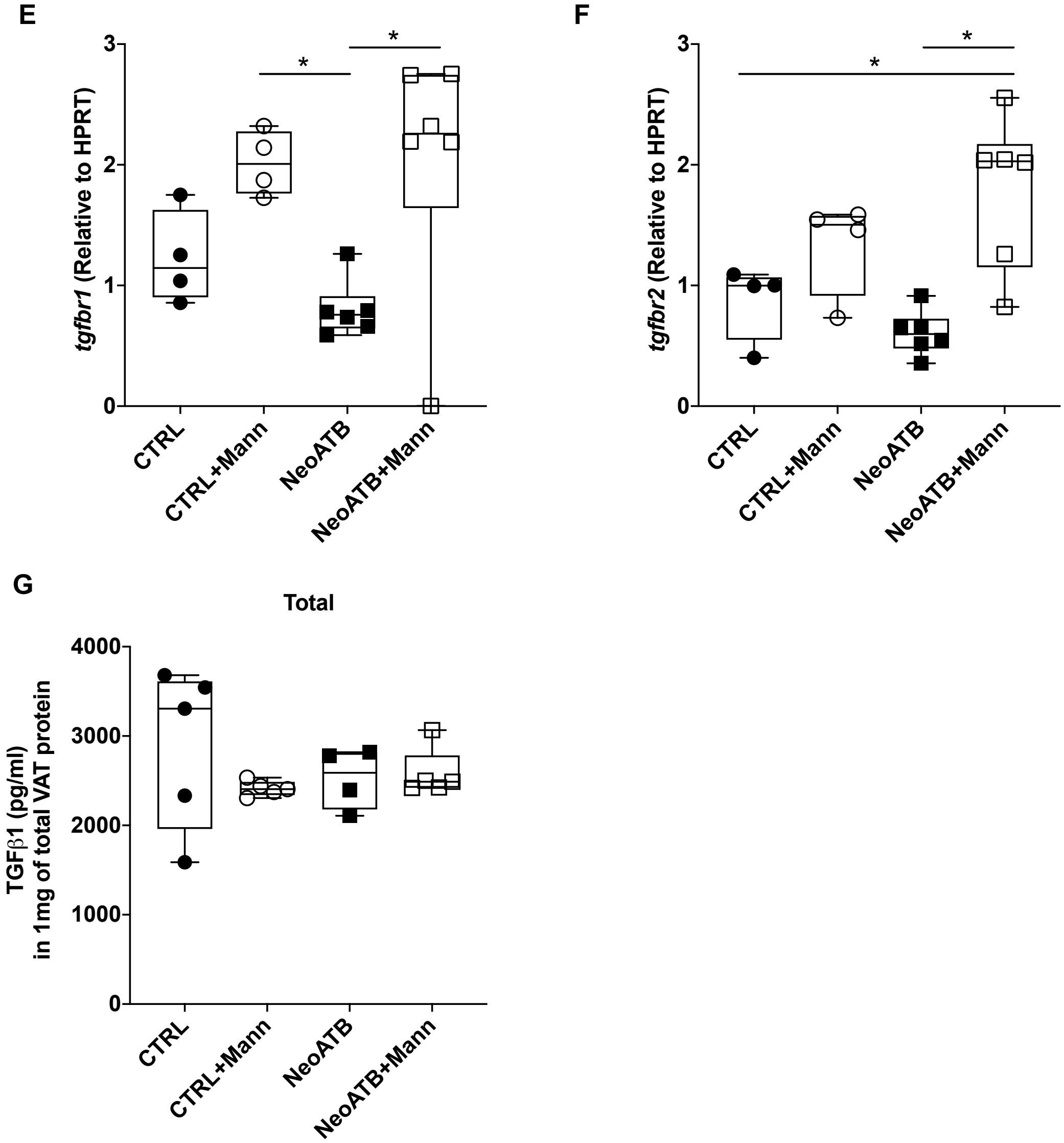

D-mannose treatment increases the proliferation of adipose Treg cells.

A. Representative FACS plots showing the frequency of $Ki67^{+}Foxp3^{+}$ (Treg) or $Ki67^{+}Foxp^{-}$ (Responder T) cells.
B. Summarized data showing the ratio of $CD4^{+}Ki67^{+}Foxp3^{+}$ to Control cells or the ratio of $CD4^{+}Ki67^{+}Foxp3^{-}$ responder T cells.
C. Summarized data showing gene expression of Interleukin-33 (*il33*) in the adipose tissue among different groups.
D. Flow cytometric analysis of CD45-CD31-SCA1+PDGFR+ VAT mesenchymal cells, which represent the primary source of IL-33 in the VAT.
E. Summarized data showing gene expression of *tgfbr1* in the adipose tissue among different groups.
F. Summarized data showing gene expression of *tgfbr2* in the adipose tissue among different groups.
G. Summarized data showing total concentration of total adipose TGFβ1 protein normalized to 1 mg of total fat protein, among different groups determined by ELISA.

Data are pooled from 2-3 independent experiments (a, b, c, e, f, g) or 1 experiment (d). Each point in the figures represents one individual animal. Statistical analysis was performed using one-way ANOVA (* $p<0.05$; ** $p<0.01$; *** $p<0.001$).

Supplemental Figure 4

(which was not certified by peer review) is the author/funder. This article is a US Government work. It is not subject to copyright under 17 USC 105 and is also made available for use under a CC0 license.

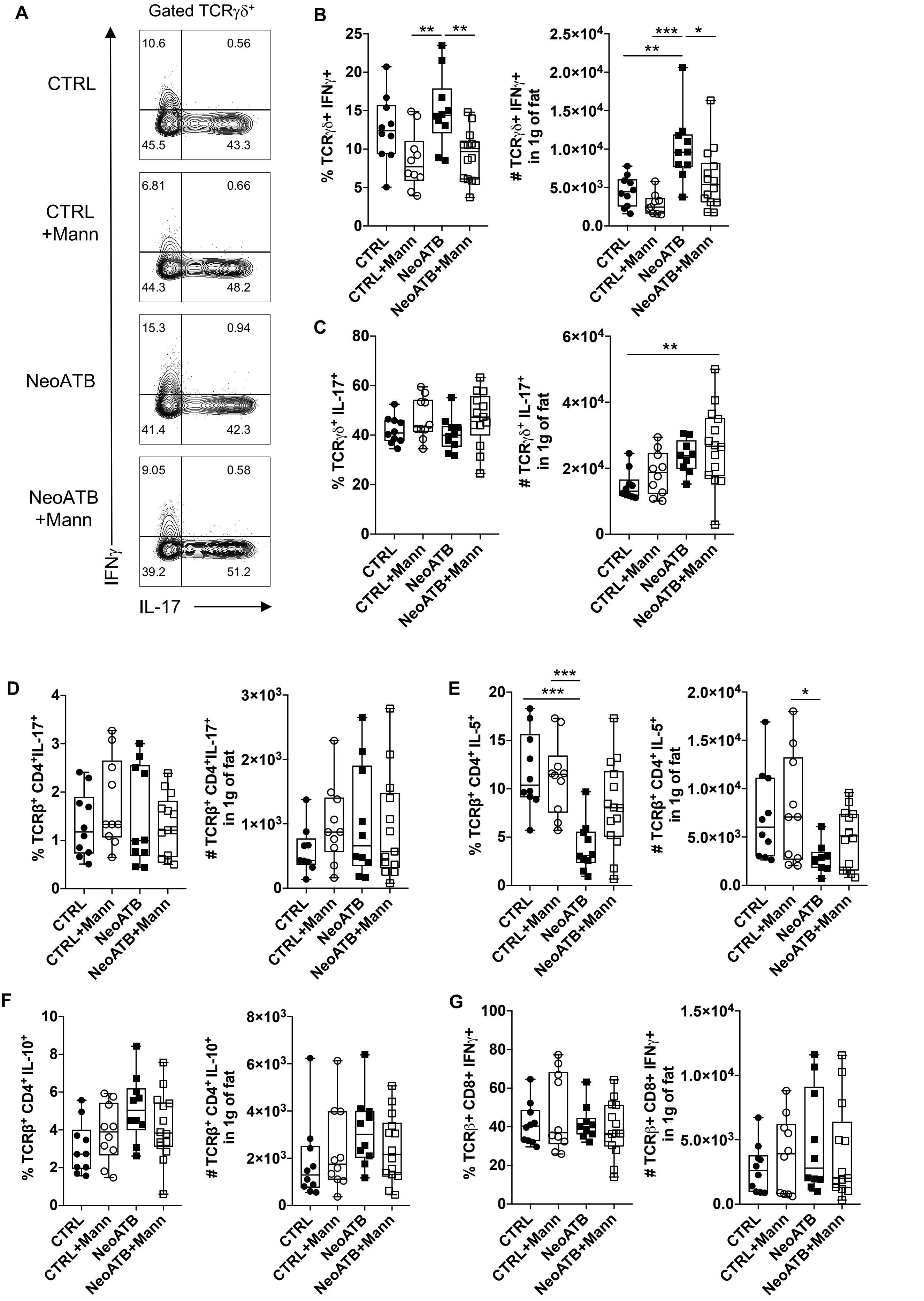

Supplemental Figure 5

(which was not certified by peer review) is the author/funder. This article is a US Government work. It is not subject to copyright under 17 USC 105 and is also made available for use under a CC0 license.

D-mannose decreases frequency and total number of adipose $TCR\gamma\delta^+IFN\gamma^+$ cells in NeoATB mice.

A. Representative FACS plots showing frequency of adipose $TCR\gamma\delta^+IFN\gamma^+$ or $TCR\gamma\delta^+IL\text{-}17^+$ cells (Gated as: $CD45^+TCR\gamma\delta^+$) among different groups.
B. Summarizing data showing frequency and total number of adipose $TCR\gamma\delta^+IFN\gamma^+$ cells among different groups.
C. Summarizing data showing frequency and total number of adipose $TCR\gamma\delta^+IL\text{-}17^+$ cells among different groups.
D. Summarizing data showing frequency and total number of adipose $CD4^+IL\text{-}17^+$ cells among different groups.
E. Summarizing data showing frequency and total number of adipose $CD4^+IL\text{-}5^+$ cells among different groups.
F. Summarizing data showing frequency and total number of adipose $CD4^+IL\text{-}10^+$ cells among different groups.
G. Summarizing data showing frequency and total number of adipose $CD8^+IFN\gamma^+$ cells among different groups.

Data are polled of 2-3 independent experiments. Each point in figures represents one individual animal. Statistical analysis was performed using one-way ANOVA (* $p<0.05$; ** $p<0.01$; *** $p<0.001$).

Supplemental Figure 5

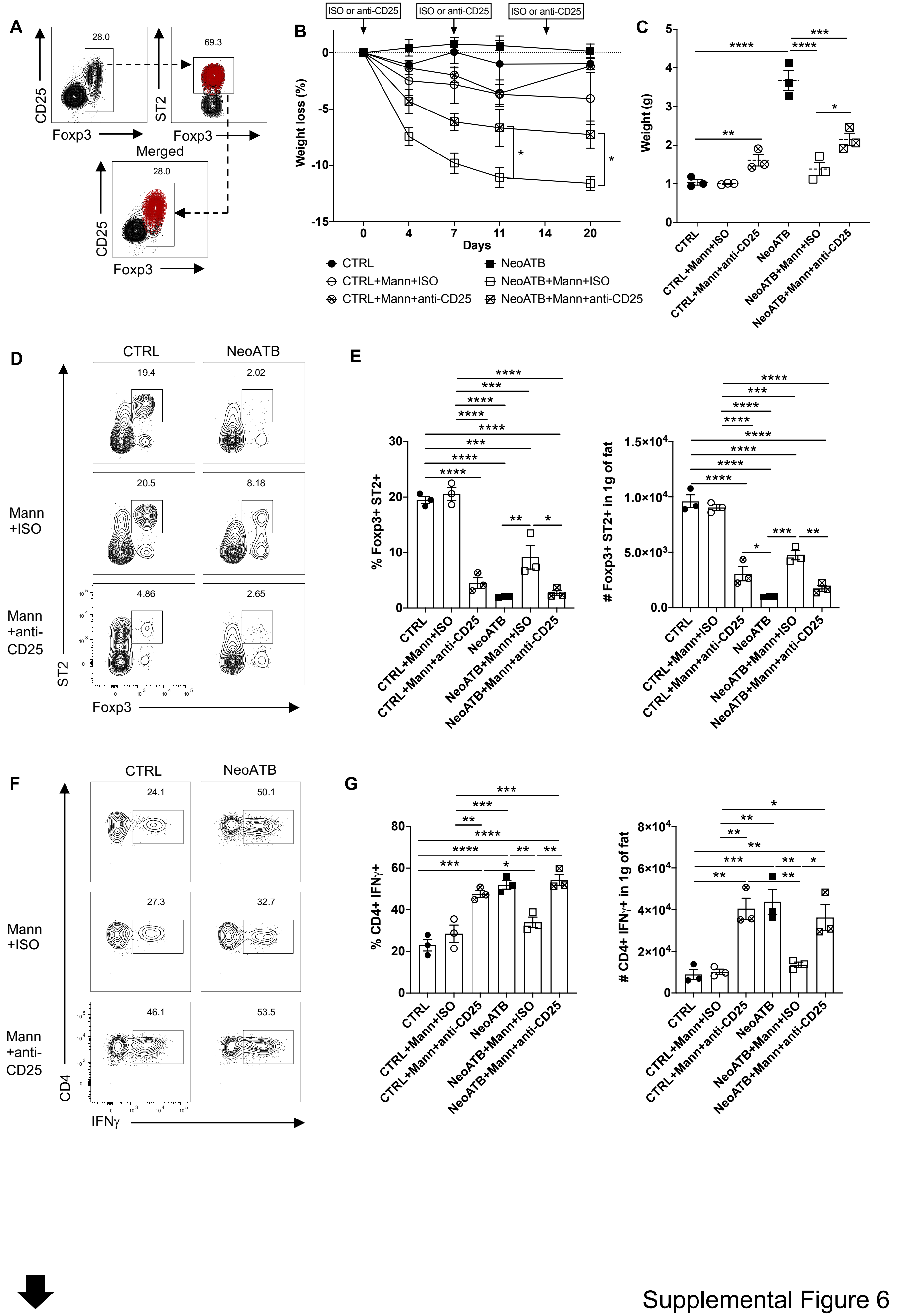

Supplemental Figure 6

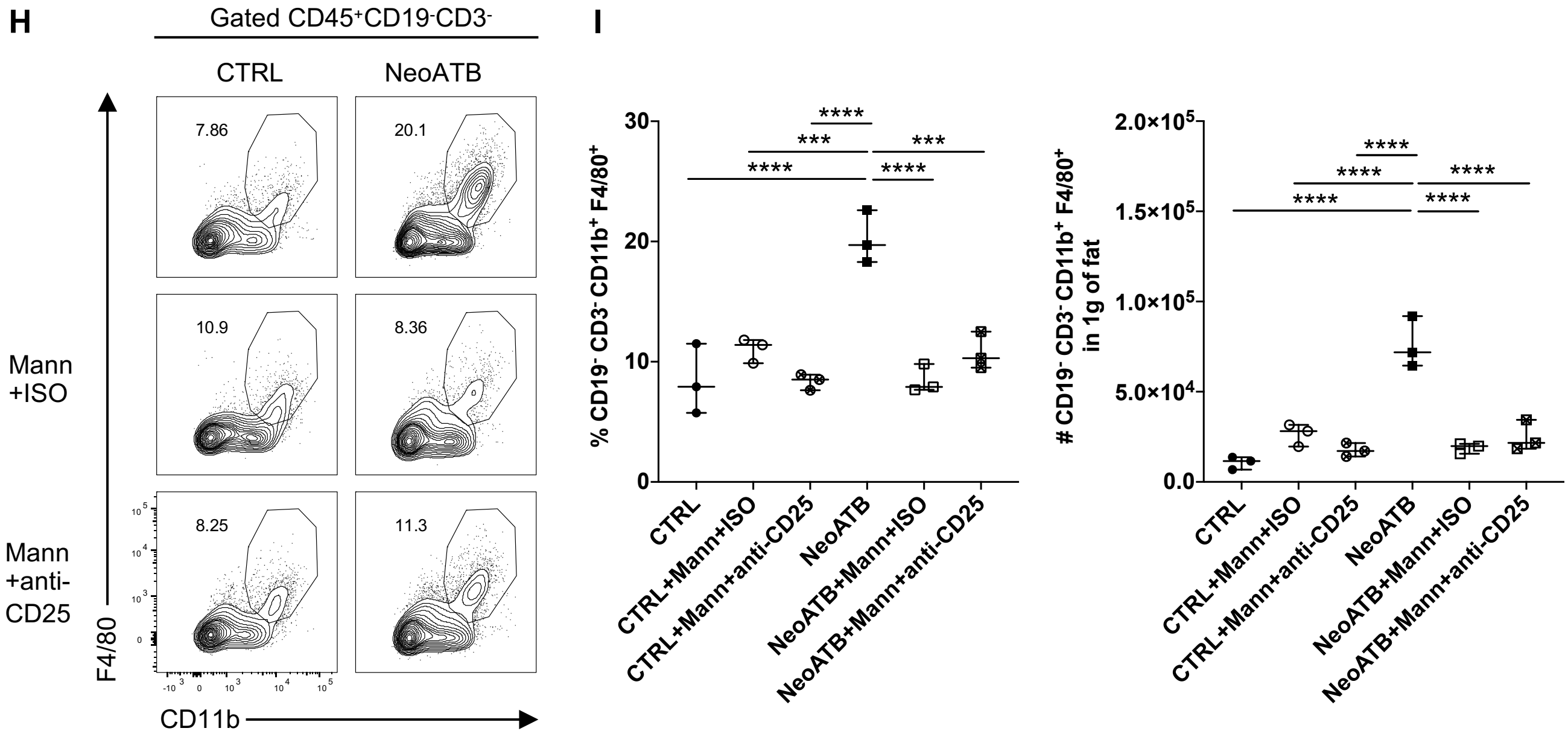

Treg depletion diminished the effect of D-mannose on the reduction of obesity in NeoATB mice.

A. Representative FACS plot showing co-expression of CD25 and ST2 among adipose Foxp3+ cells.
B. Adult aged (44-46 weeks old) CTRL and NeoATB mice were treated with/without D-Mannose or with Isotype or anti-CD25 neutralizing antibody. Weight loss (%) among different groups is shown.
C. Weight of visceral adipose tissue measured at the end of experiment among different groups.
D. Representative FACS plots showing frequency of adipose Treg cells (Gated as: $CD45^+TCR\beta^+CD4^+Foxp3^+ST2^+$) among different groups.
E. Summarizing data showing frequency and total number of adipose $Foxp3^+ST2^+$ Treg cells among different groups.
F. Representative FACS plots showing frequency of adipose Th1 $IFN\gamma^+$ cells (Gated as: $CD45^+TCR\beta^+CD4^+IFN\gamma^+$) among different groups.
G. Summarizing data showing frequency and total number of adipose Th1 $IFN\gamma^+$ cells among different groups.
H. Representative FACS plots showing frequency of adipose macrophages (Gated as: $CD45^+CD19^-CD3^-CD11b^+F4/80^+$) among different groups.
I. Summarizing data showing frequency and total number of adipose infiltrating macrophages among different groups.

Data are representative of 1 experiment (3 mice per cage/treatment). Statistical analysis was performed using one-way ANOVA (* p<0.05; ** p<0.01; *** p<0.001; **** p<0.0001). Statistical analysis in b) was performed using two-way ANOVA (* p<0.05).

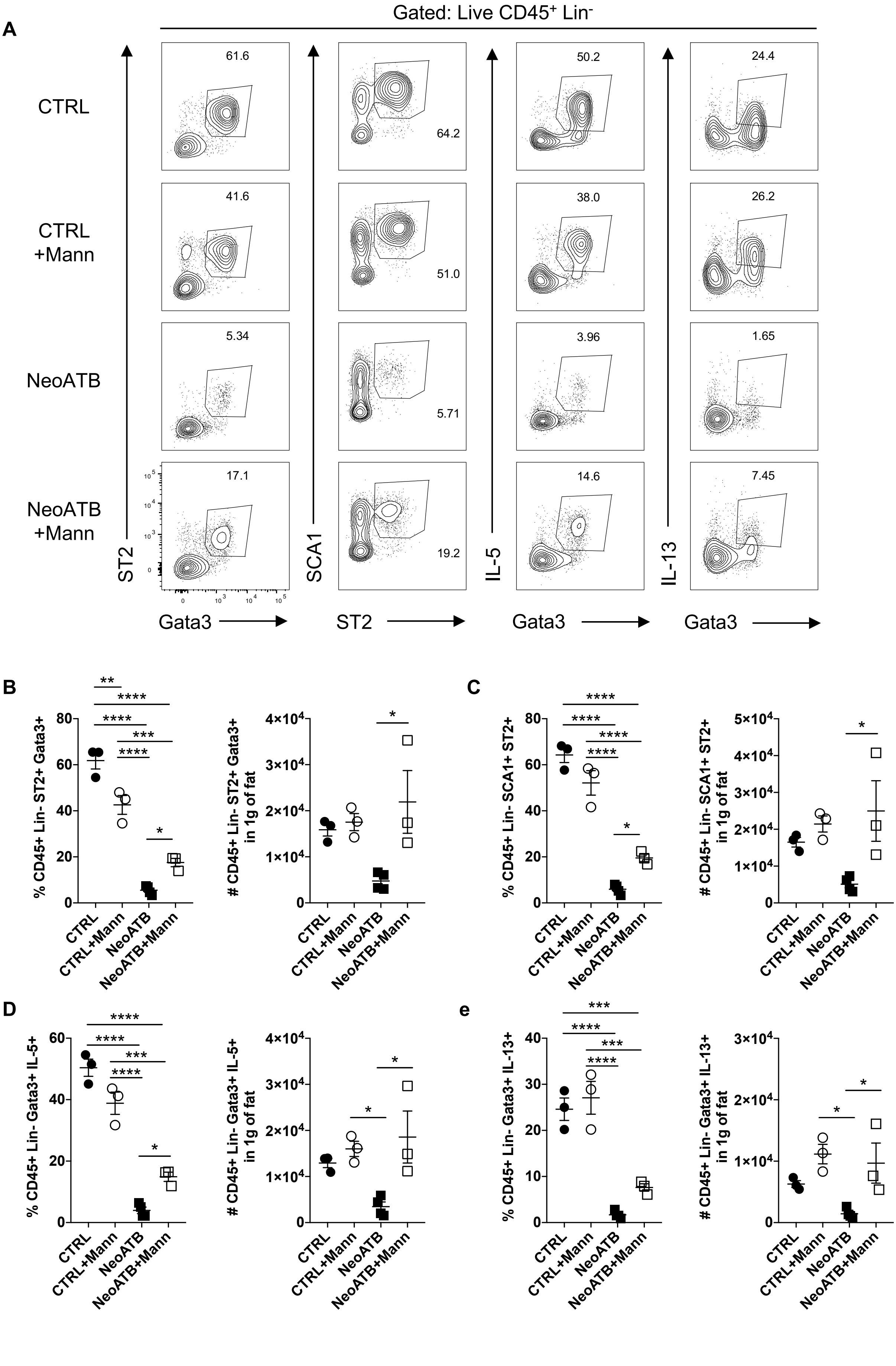

Supplemental Figure 7

D-mannose increases adipose ILC2 cells in NeoATB mice.

A. Representative FACS plots showing frequency of adipose ILC2 cells (Gated as: $CD45^{+}Lineage^{-}ST2^{+}Gata3^{+}$ or $CD45^{+}Lineage^{-}ST2^{+}SCA1^{+}$) among different groups. Representative FACS plots showing frequency of total $IL\text{-}5^{+}$ or $IL\text{-}13^{+}$ adipose ILC2 cells respectively (Gated as: $CD45^{+}Lineage^{-}Gata3^{+}IL\text{-}5^{+}$ or $CD45^{+}Lineage^{-}Gata3^{+}IL\text{-}13^{+}$).
B. Summarizing data showing frequency and total number of adipose $CD45^{+}Lineage^{-}ST2^{+}Gata3^{+}$ ILC2 cells among different groups.
C. Summarizing data showing frequency and total number of adipose $CD45^{+}Lineage^{-}ST2^{+}SCA1^{+}$ ILC2 cells among different groups.
D. Summarizing data showing frequency and total number of adipose $CD45^{+}Lineage^{-}Gata3^{+}IL\text{-}5^{+}$ ILC2 cells among different groups.
E. Summarizing data showing frequency and total number of adipose $CD45^{+}Lineage^{-}Gata3^{+}IL\text{-}13^{+}$ ILC2 cells among different groups.

Data are representative of 1 out of 2 independent experiments (3-4 mice per cage/treatment). Statistical analysis was performed using one-way ANOVA (* $p<0.05$; ** $p<0.01$; *** $p<0.001$; **** $p<0.0001$).

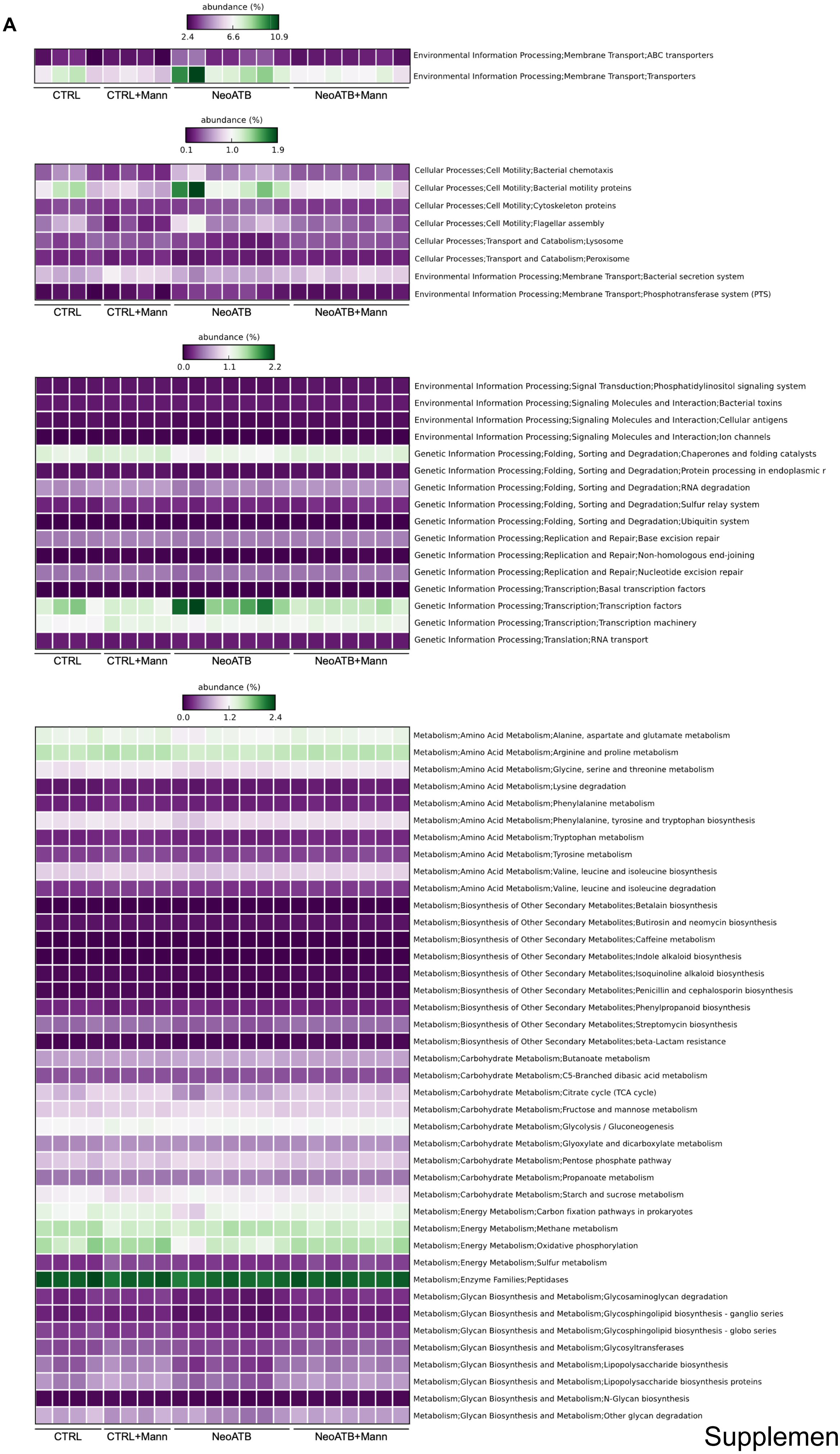

Supplemental Figure 8

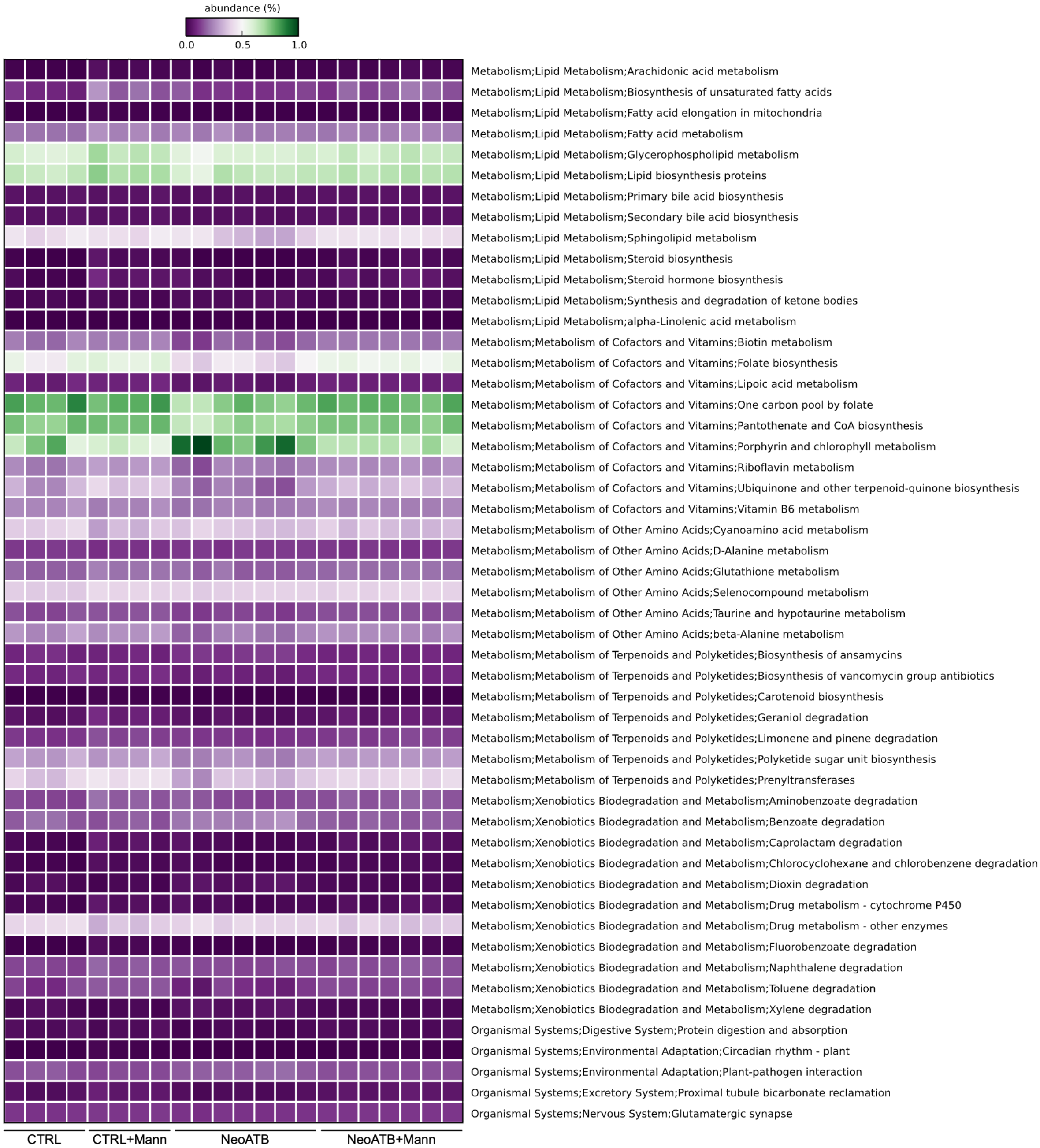

Comparative prediction of the functional metagenome of the gut microbiota.

The figure shows a graphic representation of the significant predicted metabolic pathways using PICRUSt by analysis of the corresponding OTU table generated by QIIME for the bacterial communities.

A. Heatmap plot showing all significant pathways (% of relative frequency of gene content prediction) among all groups identified by PICRUSt.
B. Box-plot figures showing representative futures of the functional metagenome predictions. The Y-axis shows the relative frequencies of gene content prediction, and the X-axis shows the different groups.

Significant differences were identified using One-way ANOVA test, followed by FDR (Benjamini Hochberg) posttest for multiple test correction.

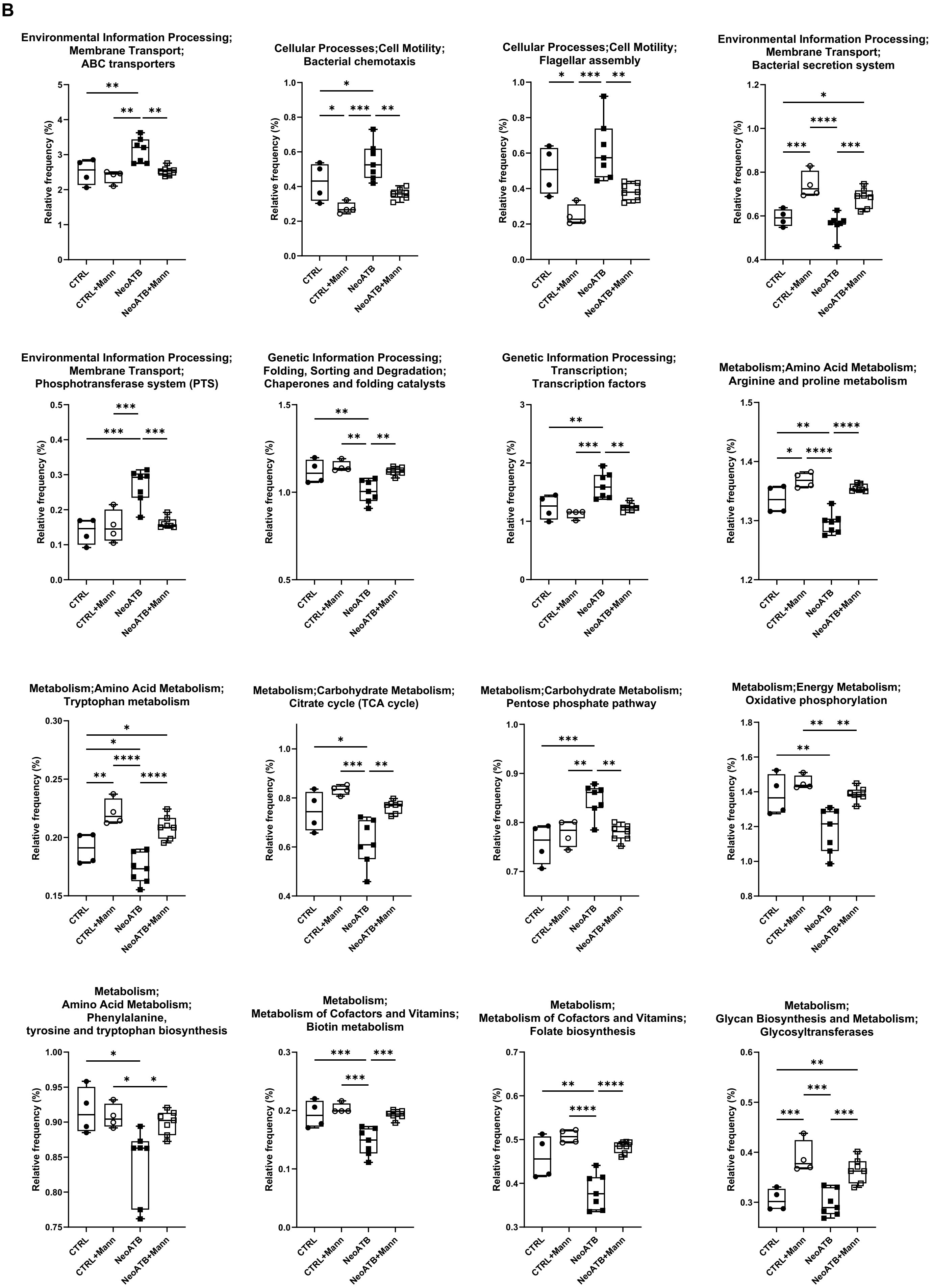

Supplemental Figure 8

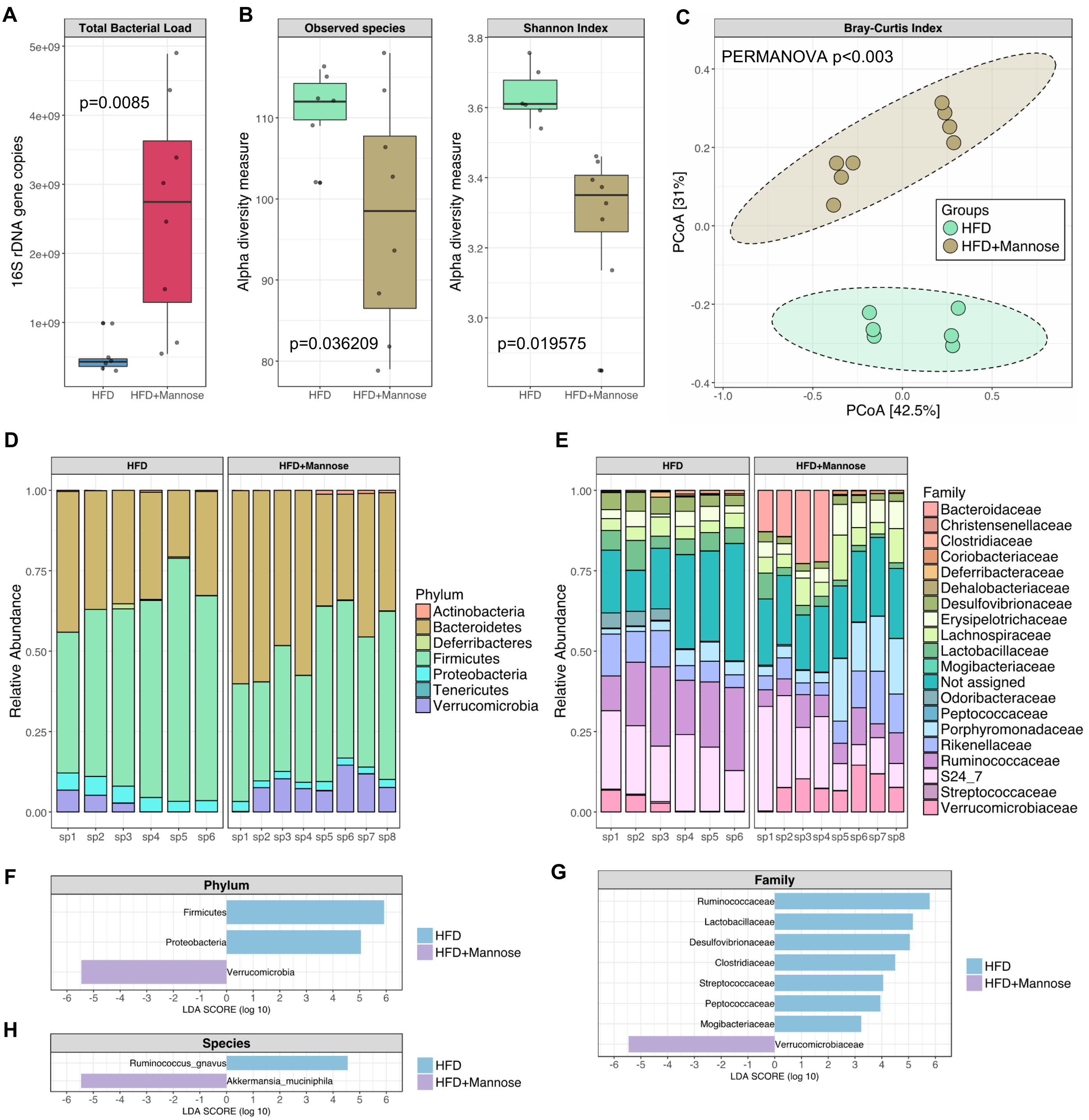

D-mannose corrects the microbiome of HFD treated mice.

A. Total bacterial load determined as number of 16S rDNA gene copies using qPCR.
B. Alpha diversity measure (Observed species, Shannon Index) between HFD and D-mannose treated HFD mice.
C. Beta diversity measure (Bray-Curtis Index) between HFD and D-mannose treated HFD mice.
D. Phylum level taxa abundance between HFD and D-mannose treated HFD mice.
E. Family level taxa abundance between HFD and D-mannose treated HFD mice.
F. Linear Discriminant Analysis (LDA=3) showing significantly different taxa on the phylum level determined by LEfSe analysis.
G. Linear Discriminant Analysis (LDA=3) showing significantly different taxa on the family level determined by LEfSe analysis.
H. Linear Discriminant Analysis (LDA=3) showing significantly different taxa on the species level determined by LEfSe analysis.

Supplemental Figure 9

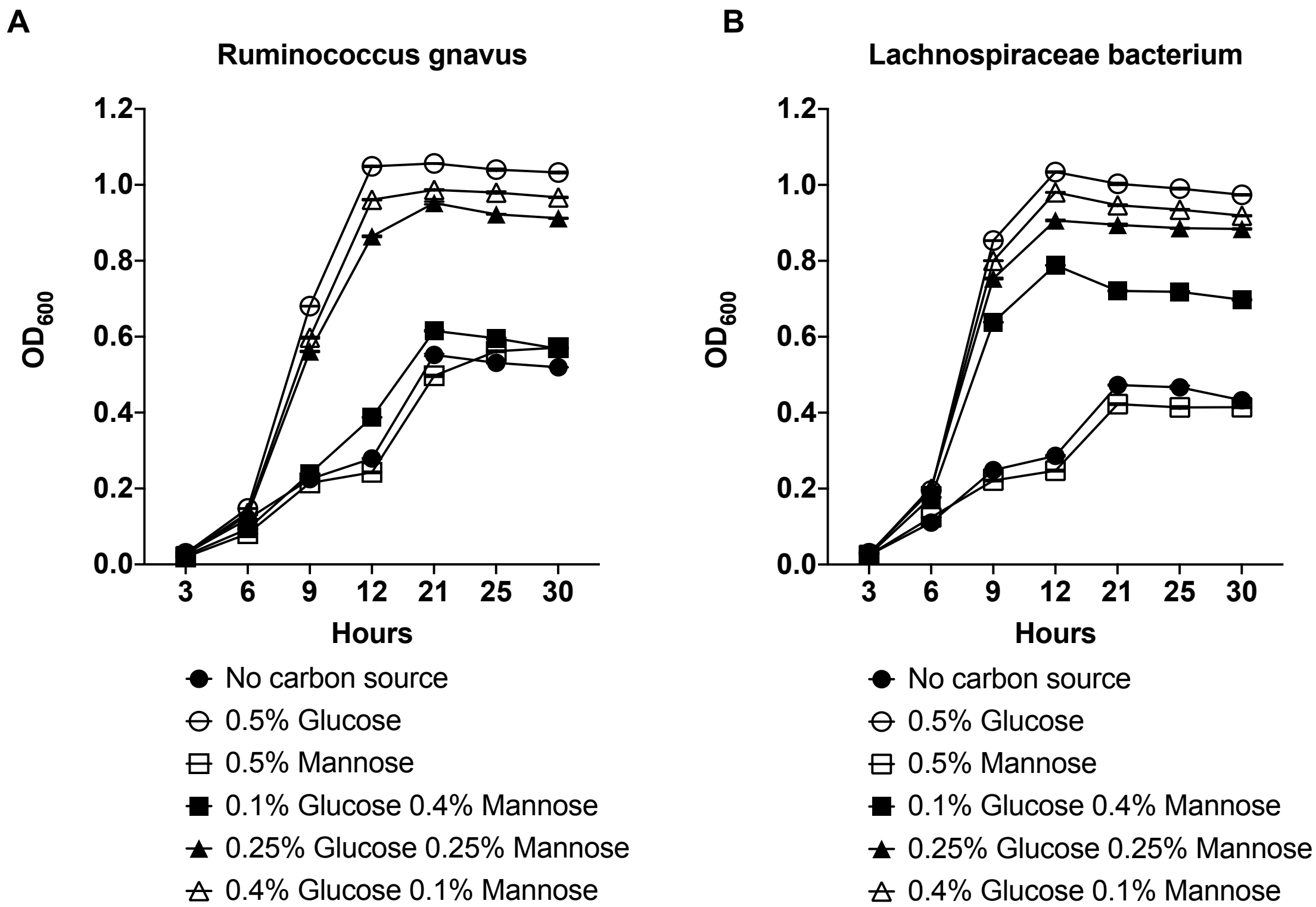

D-mannose suppresses growth of *Firmicutes*.

A. Representative growth curve of *Ruminococcus gnavus* cultured in the heart infusion media without sugar (no carbon source) or different concentration ratios of D-Mannose : D-Glucose.
B. Representative growth curve of *Lachnospiraceae bacterium* cultured in the heart infusion media without sugar (no carbon source) or different concentration ratios of D-Mannose : D-Glucose.

Data are representative of one out of 3 independent experiments.

Supplemental Table 1. Antibody used for Flow cytometry

Antibody	Fluorochrome	Clone	Catalog number	Company
Anti-mouse CD45	Alexa Fluor 700	30-F11	56-0451-82	Thermo Fisher Scientific
Anti-mouse CD4	FITC	RM4-5	11-0042-85	Thermo Fisher Scientific
Anti-mouse CD4	Brilliant Violet 510	RM4-5	100559	Biolegend
Anti-mouse CD25	PerCP-Cy5.5	PC61.5	45-0251-82	Thermo Fisher Scientific
Anti-mouse CD25	eFluor660	eBio7D4	50-0252-82	Thermo Fisher Scientific
Anti-mouse TCRβ	APC-eFluor 780	H57-597	47-5961-82	Thermo Fisher Scientific
Anti-mouse Foxp3	eFluor450	FJK-16s	48-5773-82	Thermo Fisher Scientific
Anti-mouse ST2	APC	RMST2-2	17-9335-82	Thermo Fisher Scientific
Anti-mouse Ki67	PE-eFluor610	SolA15	61-5698-82	Thermo Fisher Scientific
Anti-mouse TCRγδ	PerCP-eFluor 710	eBioGL3	46-5711-82	Thermo Fisher Scientific
Anti-mouse IFNγ	eFluor450	XMG1.2	48-7311-82	Thermo Fisher Scientific
Anti-mouse IL-17A	APC	eBio17B7	17-7177-81	Thermo Fisher Scientific
Anti-mouse CD8b	PE-Cy7	eBioH35-17.2	25-0083-82	Thermo Fisher Scientific
Anti-mouse Gata3	PE-Cy7	TWAJ	25-9966-42	Thermo Fisher Scientific
Anti-mouse SCA1	FITC	D7	11-5981-81	Thermo Fisher Scientific
Anti-mouse IL-5	PE	TRFK5	12-7052-81	Thermo Fisher Scientific
Anti-mouse IL-13	PE-eFluor610	eBio13A	61-7133-82	Thermo Fisher Scientific
Anti-mouse CD3e	APC-eFluor780	17A2	47-0032-82	Thermo Fisher Scientific
Anti-mouse CD19	PerCP-Cy5.5	eBio1D3	45-0193-82	Thermo Fisher Scientific
Anti-mouse CD11b	eFluor450	M1/71	48-0112-82	Thermo Fisher Scientific
Anti-mouse F4/80	PE-eFluor610	BM8	61-4801-82	Invitrogen
Zombie Yellow™ Fixable Viability Kit	QDOT605	N/A	423104	Biolegend
CD16/32Fc	N/A	93	14-0161-86	Thermo Fisher Scientific

Supplemental Table 2. TaqMan Assays used for qPCR

Name	Gene	Catalog number	Company
Interferon gamma	*Ifng*	Mm01168134_m1	Thermo Fisher Scientific
Interleukin 1 beta	*Il-1b*	Mm00434228_m1	Thermo Fisher Scientific
Interleukin 6	*Il-6*	Mm00446190_m1	Thermo Fisher Scientific
Interleukin 12	*Il-12*	Mm01288989_m1	Thermo Fisher Scientific
Interleukin 33	*Il-33*	Mm00505403_m1	Thermo Fisher Scientific
Tumor necrosis factor	*Tnf*	Mm00443258_m1	Thermo Fisher Scientific
TGFb receptor I	*Tgfbr1*	Mm_00436964_m1	Thermo Fisher Scientific
TGFb receptor II	*Tgfbr2*	Mm_00436976_m1	Thermo Fisher Scientific
Fatty acid binding protein 4	*Fabp4*	Mm_00445878_m1	Thermo Fisher Scientific
Free fatty acid receptor 2	*Ffar2*	Mm_02620654_s1	Thermo Fisher Scientific
Free fatty acid receptor 3	*Ffar3*	Mm_02621638_s1	Thermo Fisher Scientific
Hypoxia inducible factor 1	*Hif1a*	Mm00468869_m1	Thermo Fisher Scientific
Insulin receptor	*Insr*	Mm01211875_m1	Thermo Fisher Scientific
Glucose transporter 4	*Slc2a4*	Mm00436615_m1	Thermo Fisher Scientific
Hypoxanthine guanine phosphoribosyl transferase	*Hprt*	Mm00446968_m1	Thermo Fisher Scientific

Supplemental Table 3. Antibody used for Western blot

Antibody	Catalog number	Dilution	Company
Insulin receptor alpha	bs-0047R-TR	1:300	Bioss Antibodies
Glut4	bs-0384R-FR	1:300	Bioss Antibodies
HIF-1 Alpha	bs-0737R-TR	1:200	Bioss Antibodies
Anti-α-Tubulin	T5168-100UL	1:50000	Sigma

Supplemental Table 4. Reagents used for Seahorse Assay

Reagent	Catalog number	Company
Poly-L-Lysine	25988-63-0	MP biomedicals
RPMI medium	103576-100	Agilent technologies
Glucose	G6152-1kg	Sigma
L-Glutamine	25030164	Gibco
Pyruvate	13-115E	Lonza
Oligomycin	75351-5mg	Sigma
CCCP	C2920-10mg	Sigma
Rotenone	R8875-1g	Sigma
Antimycin A	A8674-25mg	Sigma

Supplemental Table 5. Instruments

Instrument	Company
BD LSRFortessa	BD Biosciences
BD FACSAria III Cell Sorter	BD Biosciences
Leica Aperio Scan Scope	Leica Biosystems
Seahorse XFe96 Analyzer	Agilent
SpectraMax Plus 384 Microplate Reader	Molecular devices
Accu-Chek Aviva Blood Glucometer	Accu Chek
Whitley A35 Workstation	Don Whitley Scientific
Applied Biosystems 7500 Real Time PCR System	Applied Biosystems by Thermo Fisher Scientific
QuantStudio 3	Applied Biosystems by Thermo Fisher Scientific

Supplemental Table 6. Software

Software	Company	Web
FlowJo 10	BD Biosciences	https://www.flowjo.com/solutions/flowjo
Prism 8	GraphPad	https://www.graphpad.com/scientific-software/prism/
Aperio Image Scope	Leica Biosystems	https://www.leicabiosystems.com/digital-pathology/manage/aperio-imagescope/
AdipoCount	n/a	http://www.csbio.sjtu.edu.cn/bioinf/AdipoCount/
STAMP v2.1.3	n/a	https://beikolab.cs.dal.ca/software/STAMP
